# Asymmetric Dimethylarginine at Sea Level Is a Predictive Marker of Hypoxic Pulmonary Arterial Hypertension at High Altitude

**DOI:** 10.3389/fphys.2019.00651

**Published:** 2019-05-27

**Authors:** Patricia Siques, Julio Brito, Edzard Schwedhelm, Eduardo Pena, Fabiola León-Velarde, Juan José De La Cruz, Rainer H. Böger, Juliane Hannemann

**Affiliations:** ^1^ Institute of Health Studies, Universidad Arturo Prat, Iquique, Chile; ^2^Institute DECIPHER, German-Chilean Institute for Research on Pulmonary Hypoxia and its Health Sequelae, Hamburg, Germany and Iquique, Chile; ^3^ Institute of Clinical Pharmacology and Toxicology, University Medical Center Hamburg-Eppendorf, Hamburg, Germany; ^4^ Department of Biological and Physiological Sciences, Facultad de Ciencias y Filosofía, Universidad Peruana Cayetano Heredia, Lima, Peru; ^5^ Department of Preventive Medicine and Public Health, Universidad Autónoma de Madrid, Madrid, Spain

**Keywords:** ADMA, SDMA, nitric oxide, hypobaric hypoxia, right ventricle, endothelium, risk prediction

## Abstract

**Background:** Prolonged exposure to altitude-associated chronic hypoxia (CH) may cause high-altitude pulmonary hypertension (HAPH). Chronic intermittent hypobaric hypoxia (CIH) occurs in individuals who commute between sea level and high altitude. CIH is associated with repetitive acute hypoxic acclimatization and conveys the long-term risk of HAPH. As nitric oxide (NO) regulates pulmonary vascular tone and asymmetric dimethylarginine (ADMA) is an endogenous inhibitor of NO synthesis, we investigated whether ADMA concentration at sea level predicts HAPH among Chilean frontiers personnel exposed to 6 months of CIH.

**Methods:** In this prospective study, 123 healthy army draftees were subjected to CIH (5 days at 3,550 m, 2 days at sea level) for 6 months. In 100 study participants with complete data, ADMA, symmetric dimethylarginine (SDMA), L-arginine, arterial oxygen saturation (SaO_2_), systemic blood pressure, and hematocrit were assessed at months 0 (sea level), 1, 4, and 6. Acclimatization to altitude was determined using the Lake Louise Score (LLS) and the presence of acute mountain sickness (AMS). Echocardiography was performed after 6 months of CIH in 43 individuals with either good (*n* = 23) or poor (*n* = 20) acclimatization.

**Results:** SaO_2_ acutely decreased at altitude and plateaued at 90% thereafter. ADMA increased and SDMA decreased during the study course. The incidence of AMS and the LLS was high after the first ascent (53 and 3.1 ± 2.4) and at 1 month of CIH (47 and 3.0 ± 2.6), but decreased to 20 and 1.4 ± 2.0 at month 6 (both *p* < 0.001). Eighteen participants (42%) showed a mean pulmonary arterial pressure (mPAP) >25 mm Hg, out of which 9 (21%) were classified as HAPH (mPAP ≥ 30 mm Hg). ADMA at sea level was significantly associated with mPAP at high altitude in month 6 (*R* = 0.413; *p* = 0.007). In ROC analysis, a cutoff for baseline ADMA of 0.665 μmol/L was determined to predict HAPH (mPAP > 30 mm Hg) with a sensitivity of 100% and a specificity of 63.6%.

**Conclusions:** ADMA concentration increases during CIH. ADMA at sea level is an independent predictive biomarker of HAPH. SDMA concentration decreases during CIH and shows no association with HAPH. Our data support a role of impaired NO-mediated pulmonary vasodilation in the pathogenesis of HAPH.

## Introduction

Global hypobaric hypoxia as it occurs in high altitude is a major stressor for cardiorespiratory physiology. In populations living under conditions of chronic hypobaric hypoxia (CH) in different regions of the world, diverse biological mechanisms to adapt to high altitude-associated hypoxic conditions have evolved (Simonson, 2015). Acute exposition to high altitude may cause acute mountain sickness (AMS), a condition involving headache, dizziness, anorexia, and nausea (Barry and Pollard, 2003). In addition, exposition to hypoxia is associated with acute elevation of pulmonary arterial pressure and – chronically – with remodeling of the right ventricle which may ultimately lead to heart failure (Leon-Velarde et al., 2005; Penaloza and Arias-Stella, 2007). Therefore, high altitude-associated pulmonary hypertension (HAPH) is a severe health consequence of chronic exposure to hypoxia (Grimminger et al., 2017) with a prevalence of up to 15% (Leon-Velarde et al., 2005). In individuals who commute regularly between high altitude and sea level, acclimatization to altitude conditions is repetitive. This type of exposition is called chronic intermittent hypobaric hypoxia (CIH) and has been acknowledged as a separate condition (Richalet et al., 2002; Brito et al., 2007, 2018). Like chronic hypoxia, CIH may lead to (mild) HAPH (Brito et al., 2018); while AMS may occur after each reascent to high altitude, albeit with decreasing prevalence (Richalet et al., 2002).

In understanding the pathophysiology of HAPH, multiple mechanisms have been discussed [for review, cf. (Grimminger et al., 2017)]. Nitric oxide (NO), a major endothelial vasodilator mediator, is upregulated during hypoxia in the systemic circulation, where hypoxia causes vasodilation (Blitzer et al., 1996). We hypothesized that hypoxic vasoconstriction in the pulmonary circulation may be caused or aggravated by reduced NO production (Kylhammar and Radegran, 2017). Our hypothesis was based on observations in animal models and in man showing that the levels of asymmetric dimethylarginine (ADMA), an endogenous, competitive inhibitor of NO synthesis [for review, cf. (Böger, 2006)], are increased in hypoxia. Our group previously reported that ADMA plasma concentration is upregulated in healthy young individuals during their first exposure to CIH (Lüneburg et al., 2017). In a rat model of CIH and CH, we found upregulation of plasma ADMA concentration concomitantly with downregulation of the expression of dimethylarginine dimethylaminohydrolase (DDAH), the major enzyme involved in ADMA metabolism (Lüneburg et al., 2016). More recently, we observed a prevalence of HAPH (mPAP > 30 mm Hg) of 9% in a cross-sectional survey of workers in the Andean plateau who had been exposed to CIH for a mean of 14 years (Brito et al., 2018). In these individuals, ADMA was significantly associated with mean pulmonary arterial pressure (mPAP), further suggesting a pathophysiological link between ADMA and HAPH. By contrast to ADMA, its congener, symmetric dimethylarginine (SDMA), does not directly interfere with NO synthesis (Schwedhelm and Böger, 2011).

In the present study, we prospectively investigated a large group of young, primarily healthy Chilean lowlanders who were exposed to CIH for 6 months. We investigated whether ADMA and/or SDMA are suitable as predictive markers of HAPH. For this purpose, we determined the time courses of ADMA and SDMA in plasma and performed echocardiography after 6 months of CIH in a subgroup of study participants to prospectively relate these potential prognostic biomarkers with mPAP.

## Patients and Methods

### Study Participants and Protocol

**Figure 1:**
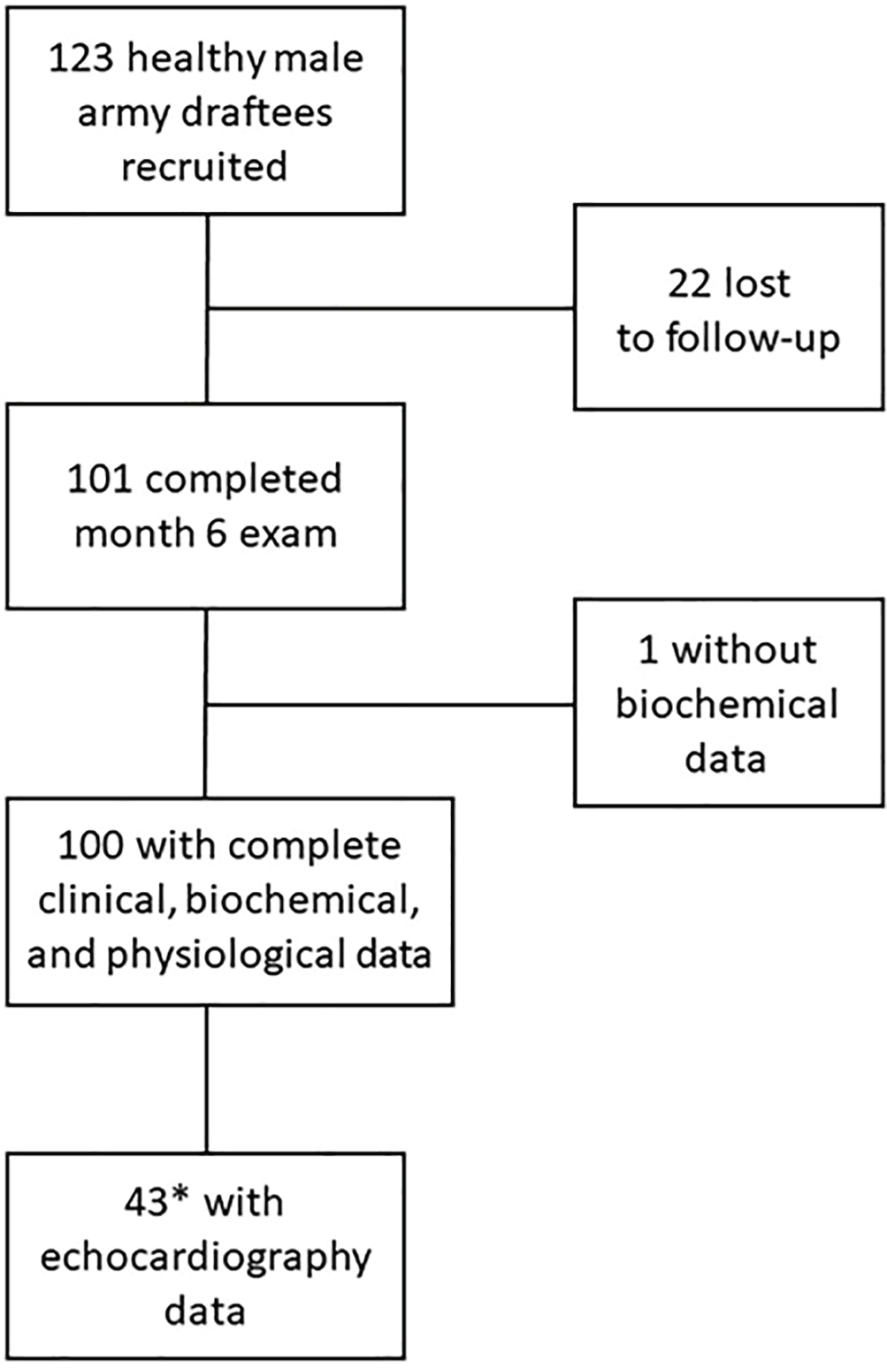
CONSORT diagram of participant flow in the study. *The subgroup for echocardiography was selected to represent participants with good and poor acclimatization to high altitude, respectively, as measured by the presence or absence of acute mountain sickness and low arterial oxygen saturation.

We included 123 male army draftees into this prospective cohort study of chronic intermittent hypobaric hypoxia (CIH). None of the study participants had previously been exposed to high altitude. Age, weight, height, and smoking habits were recorded. All study participants were declared healthy after a thorough baseline medical examination. During the study course, 22 study participants (17.9%) were lost to follow-up because of nonmedical reasons, and one subject had to be excluded from the analysis because of missing biochemical data (Figure 1); 100 study participants were included in the final statistical analysis. Echocardiography at month 6 was performed in a subgroup of 44 subjects. Out of these, 43 subjects with valid ADMA measurements at month 6 were included in the final analysis.

After the baseline investigation, the study participants adhered to a shift regimen of 5 days on duty at high altitude (HA; 3,550 m) followed by 2 days of recovery at sea level (SL) during the whole study period of 6 months. A daily routine including a defined moderate exercise regimen (half an hour of walking at high altitude or 1 h of running exercise at sea level, respectively; no strenuous exercise at high altitude) and a controlled diet (3,000 kcal daily) were maintained. Alcohol and coffee were not accessible at high altitude, drinking tea was allowed twice per day. Occasional beer consumption during off-time on weekends at sea level was reported by 57% of the study participants.

Measurements were taken on 5 predefined study days: At month 0 (M0), a baseline investigation was performed at sea level (M0 [SL]; before first exposure to high altitude) as well as at high altitude [M0 (HA)]. Further study days were scheduled at month 1 (M1), at month 4 (M4), and at month 6 (M6) of exposure to CIH. All blood samples were drawn early in the morning, after 8 h of fasting. Measurements at high altitude [M0 (HA), M1, M4, and M6] were taken on the first day of the corresponding shift, 18 h after arrival from sea level.

Written informed consent was obtained from all study participants before the start of the study. This study was approved by the Ethical Committee of Universidad Arturo Prat, Iquique, Chile.

### Variables Measured

On each study day, systolic blood pressure (SBP) and diastolic blood pressure (DBP) were determined on the right arm of each participant in sitting position after 5 min of rest, using appropriately sized cuffs and calibrated standard mercury sphygmomanometers. Heart rate (HR) and oxygen saturation (SaO_2_) were measured with a HR-100C Omron device (Omron®, Health Care Inc., Bethesda, USA) and a pulse oximeter (POX050, Mediaid®, Cerritos, USA), respectively. For all of these measurements, the mean of two readings separated by a 3-min interval was used according to international guidelines (Chobanian et al., 2003).

The Lake Louise Score (LLS), a self-assessment test describing the severity of acute mountain sickness (AMS) at altitude (Roach et al., 1993), was assessed on each of the 4 study days at high altitude; AMS was diagnosed clinically when headache and one or more other typical symptoms occurred (i.e., nausea or vomiting, insomnia, dizziness, lassitude, or fatigue) and an LLS of >3 was achieved. Severity was assessed according to the following score: mild (3–4), moderate (5–10), and severe (11–15) (Roach et al., 1993).

Venous blood samples were taken without blood stasis at M0 (SL), M1, M4, and M6 for the determination of hematocrit, hemoglobin, L-arginine, ADMA, and SDMA. Hematocrit and hemoglobin were measured on the day of blood withdrawal using an automatic hematological counter (Cell-dyn 3700®, Tecnigen, Santiago, Chile). Blood was centrifuged and EDTA plasma kept frozen at −20°C until analysis of L-arginine and dimethylarginines.

### Echocardiography

Echocardiography was performed at high altitude (3,550 m) in a subgroup of 43 study participants. As in a previous study we showed a correlation of ADMA with AMS (Lüneburg et al., 2017), selection was based upon SaO_2_ at month 6 plus the presence or absence of AMS at 24 h after reascent to altitude in month 6. Good acclimatization was defined as absence of AMS plus SaO_2_ > 89% and poor acclimatization was defined as presence of AMS and/or SaO_2_ < 89%, 20 study participants showed a poor and 23 a good response to high altitude, respectively.

Right heart measurements were obtained in a sanitary facility 24 h after ascent to altitude, using an echocardiograph (GE Vivid-I®, GE Healthcare Systems, Tirat Carmel, Israel) with a 1.5–3.6 MHz phased array probe after aligning the tip of ultrasound beam at true left ventricular apex. All echocardiographical measurements were performed in compliance with AHA guidelines (Rudski et al., 2010). Pulmonary ejection time and pulmonary acceleration times were obtained with pulse Doppler recordings of the pulmonary valve from a parasternal short axis view at aortic valve level, and tricuspid gradient was measured. Systolic and mean pulmonary artery pressure (mPAP) were calculated from pulmonary acceleration time as 4 × maximum velocity^2^ + right atrial pressure and according to Mahan formula (Dabestani et al., 1987). Additionally, pulmonary vascular resistance (PVR) was calculated as tricuspid reflux maximum velocity/velocity time integral multiplied by 10 (right atrium pressure) + 0.16. Two separate cut-off criteria were used to define the presence of pulmonary hypertension (PH): the consensus definition of high-altitude pulmonary hypertension (HAPH; mPAP > 30 mm Hg) (Leon-Velarde et al., 2005), and the general cutoff for pulmonary arterial hypertension at sea level, which is defined at mPAP > 25 mm Hg (Galie et al., 2016). The purpose of including the sea level cutoff was to have comparative panorama since CIH entails a substantial period of both, high altitude and sea level exposure.

### Analysis of L-Arginine and Dimethylarginines by Liquid Chromatography-Tandem Mass Spectrometry Method

L-Arginine, ADMA, and SDMA were quantified using a validated liquid chromatography-tandem mass spectrometry method (LC-MS/MS) as described before (Schwedhelm et al., 2007). Briefly, 25 μl of EDTA plasma was spiked with stable isotope-labeled L-arginine and ADMA as internal standards (stable isotope-labeled ADMA being used to quantify unlabeled ADMA and SDMA). Proteins were precipitated with 100 μl of methanol. After filtrating the samples through a 0.22-μm hydrophilic membrane (Multiscreen HTS™, Millipore, Molsheim, France), compounds were derivatized to their butylester derivatives with butanolic 1 N HCl, and analyzed by LC-MS/MS (Varian 1200 MS, Agilent Technologies, Santa Clara, USA). The accuracy and precision were >98% and >95% for all analytes, respectively.

### Statistical Analyses

Variables were tested for normal distribution using the Kolmogorov-Smirnov test. Differences between groups were tested for significance by using either the nonparametric Mann-Whitney U test for two groups or analysis of variance with the Kruskal-Wallis test or Dunn’s multiple comparisons test for more than two groups. Repeated measurement ANOVA with Bonferroni correction was used for time courses of plasma biomarker concentrations. Correlations were calculated using linear regression or the Spearman test. Receiver-operated curve (ROC) analyses were constructed to assess the optimal cut-off value for ADMA. All statistical analyses were performed using SPSS (version 21; IBM Corporation, Armonk, NY, USA). Data are presented as mean with standard deviation or as median with 25th and 75th percentiles, as appropriate. For all tests, *p* < 0.05 was considered significant.

## Results

### Baseline Characteristics

A total of 123 young healthy male army draftees were recruited for this study; they had a mean age of 18.3 ± 1.3 years. All study participants were normotensive and had hemoglobin and hematocrit levels as well as oxygen saturation within the normal ranges, respectively. Table 1 displays the baseline demographic and anthropometric characteristics of the initial study cohort (*n* = 123), of the 100 study participants with complete data until month 6, and of the subgroup with echocardiography data (*n* = 43). There were no significant differences between the three groups.

**Table 1:** Baseline characteristics of the study cohort.

		*n* = 123^1^	*n* = 100^2^	*n* = 43^3^
**Demographics**				
Age	years	18.3 ± 1.3	18.2 ± 1.1	18.1 ± 0.8
Smoking status	% current smokers	50.4	52.0	48.8
**Physiological and biochemical parameters**				
Height	m	1.72 ± 0.07	1.72 ± 0.08	1.70 ± 0.07
Weight	kg	72.2 ± 14.4	72.9 ± 15.0	72.3 ± 15.7
Oxygen saturation (SL)	%	98.0 ± 0.7	98.1 ± 0.7	98.1 ± 0.8
Systolic blood pressure (SL)	mm Hg	111.5 ± 10.7	111.9 ± 10.8	113.5 ± 10.3
Diastolic blood pressure (SL)	mm Hg	71.2 ± 7.9	71.5 ± 7.9	72.2 ± 7.7
Heart rate (SL)	1/min	73.5 ± 11.2	74.3 ± 11.2	75.7 ± 12.7
Hematocrit	%	45.0 ± 1.7	45.0 ± 1.7	45.0 ± 1.6
Hemoglobin	mg/dl	15.0 ± 0.8	15.0 ± 0.7	15.0 ± 0.7

1 Complete, initially recruited study cohort.

2 Study participants with complete clinical, biochemical, and physiological data.

3 Study participants with echocardiography data. Data are given as mean ± standard deviation. Abbreviations: SL, sea level.

### Effects of Exposure to Chronic Intermittent Hypobaric Hypoxia on Physiological Variables and Incidence of Acute Mountain Sickness

There was an acute drop in oxygen saturation from 98.1 ± 0.7 to 83.8 ± 5.4% immediately after the first ascent to high altitude. During the further course of the study, oxygen saturation stabilized at about 90% (Table 2). Hematocrit and hemoglobin increased during the first month of CIH and remained stable thereafter at about 47–49%. An inverse pattern of response was observed for blood pressure and heart rate, which increased from M0 (SL) to M0 (HA), but returned to baseline levels subsequently (Table 2).

**Table 2:** Time course of physiological and hematological variables during chronic intermittent hypobaric hypoxia (*n* = 100).

	M0 (SL)	M0 (HA)	M1	M4	M6	*p*
Oxygen saturation [%]	98.1 ± 0.7	83.8 ± 5.4*	90.0 ± 4.5	90.3 ± 3.6	89.6 ± 5.0	<0.001
Hematocrit [%]	45.0 ± 1.7	n.d.	47.3 ± 2.4*	49.0 ± 2.7*	48.5 ± 2.4*	<0.001
Hemoglobin [mg/dl]	15.0 ± 0.7	n.d.	15.6 ± 0.8*	16.2 ± 0.9*	16.1 ± 0.8*	<0.001
LLS	n.a.	3.1 ± 2.4	3.0 ± 2.6	1.7 ± 2.0^#^	1.4 ± 2.0^#^	<0.001
AMS [%]	n.a.	53	47	24^#^	20^#^	<0.001
Systolic blood pressure [mm Hg]	111.9 ± 10.7	118.6 ± 13.4*	100.0 ± 8.3^#^	108.7 ± 8.4^#^	108.9 ± 8.5^#^	<0.001
Diastolic blood pressure [mm Hg]	71.5 ± 7.9	73.5 ± 9.7	64.2 ± 5.8^#^	68.3 ± 7.2^#^	70.2 ± 8.3	<0.001
Heart rate [1/min]	74.3 ± 11.2	104.3 ± 18.9*	87.3 ± 13.0^#^	76.3 ± 10.6^#^	75.4 ± 9.8^#^	<0.001

All measurements were taken at high altitude, except at month 0 (M0), when separate measurements were taken at sea level (before the first ascent to high altitude; SL) and at high altitude (HA). * Statistical significance for difference versus M0 (SL); ^#^ Statistical significance for difference versus M0 (HA) with Dunn’s multiple comparisons test; *p* denotes the overall *p*-value of Kruskal-Wallis test between measurements. All data are given as mean ± standard deviation. Abbreviations: M0, month 0; M1, month 1; M4, month 4; M6, month 6; LLS, Lake Louise Score; AMS, prevalence of acute mountain sickness; n.a., not applicable; n.d, not determined.

The mean Lake Louise Score (LLS) after the first ascent to HA at M0 was 3.1 ± 2.4; 53 study participants developed AMS. The LLS as well as the incidence of AMS were still high at month 1 (3.0 ± 2.6 and 47, respectively). Thereafter, both parameters decreased to 1.4 ± 2.0 and 20, respectively, at M6 (*p* < 0.01 for both; Table 2).

### Effects of Exposure to Chronic Intermittent Hypobaric Hypoxia on L-Arginine and Dimethylarginine Concentrations

**Figure 2:**
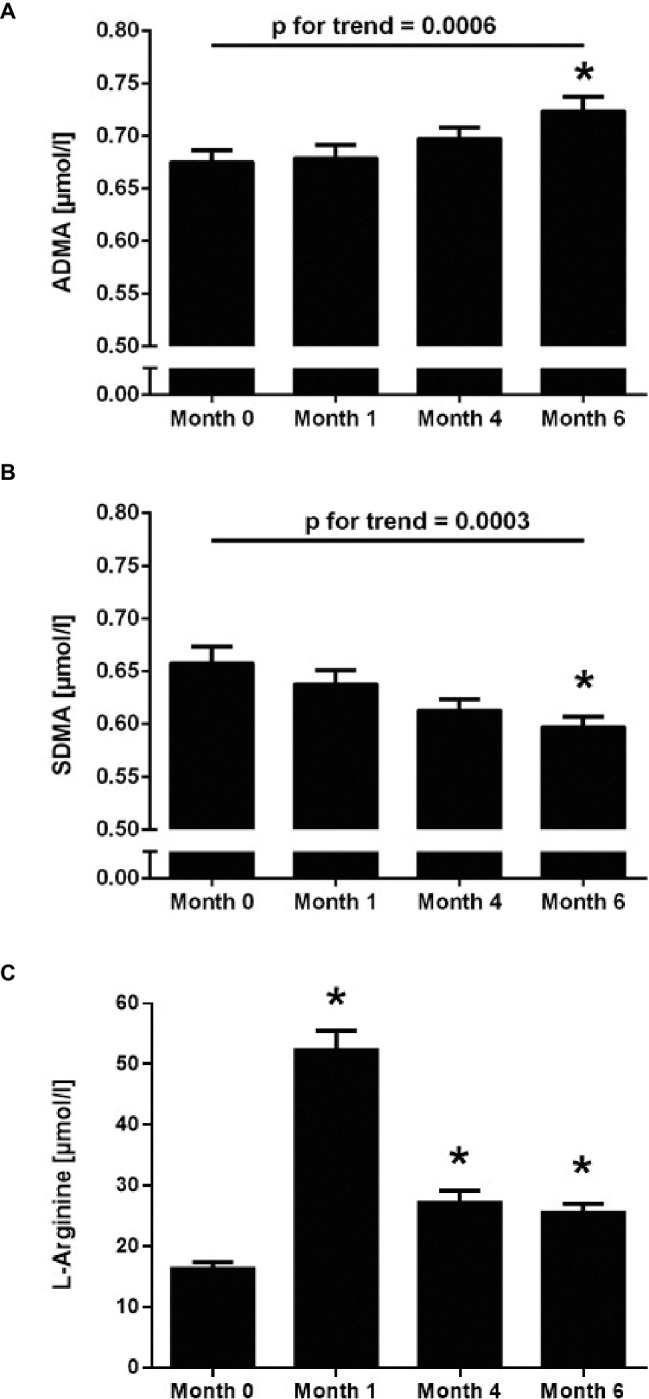
Time course of the plasma concentrations of ADMA **(A)**, SDMA **(B)**, and L-arginine **(C)** in 100 healthy young individuals at baseline sea level conditions (Month 0) and at high altitude after 1, 4, and 6 months of chronic intermittent hypobaric hypoxia. Values are mean ± standard deviation. *p < 0.01 versus Month 0; *p* for linear trend was determined by one-way ANOVA.

The baseline ADMA plasma concentration was 0.68 ± 0.11 μmol/L. There was a highly significant, continuous increase of ADMA concentration during CIH, reaching 0.73 ± 0.13 μmol/L at month 6 (*p* < 0.001 for trend; Figure 2A). By contrast, plasma SDMA concentration significantly decreased over time during CIH (*p* < 0.001 for trend; Figure 2B). L-Arginine plasma concentration was 16.5 ± 10.6 μmol/L at baseline; it significantly increased at month 1, but leveled off again at months 4 and 6 (Figure 2C).

There was a weak, but significant correlation of ADMA plasma concentration with hematocrit (*R* = 0.181, *p* < 0.01), while SDMA did not correlate with hematocrit. Neither ADMA nor SDMA plasma concentrations were significantly associated with systolic blood pressure, diastolic blood pressure, SaO_2_, the Lake Louise Score, or the incidence of AMS.

At baseline sea level conditions, ADMA concentration was significantly lower in nonsmokers than in smokers (0.65 ± 0.10 vs. 0.70 ± 0.11 μmol/L, respectively, *p* = 0.02). After 6 months of CIH, ADMA had increased in both groups and was no longer significantly different between nonsmokers and smokers (0.71 ± 0.12 vs. 0.74 ± 0.13 μmol/L, *p* = n.s.).

### Echocardiographic Assessment of Right Ventricular Function and Pulmonary Arterial Pressure

Echocardiography was performed in a subgroup of 43 study participants for whom complete data sets were available at month 6. All echocardiographic parameters were within the respective usual normal ranges, except systolic pulmonary arterial pressure and mean pulmonary arterial pressure, which were 28.3 ± 6.5 and 23.1 ± 7.5 mm Hg, respectively (Table 3). There were no significant differences in any of the parameters between well and poorly acclimatized subjects, respectively.

**Table 3:** Echocardiographic and biochemical parameters of the study group (*n* = 43).

	All	Well acclimatized	Poorly acclimatized	p*
*N*	43	23	20	
RAA [cm^2^]	13.7 ± 3.2	13.7 ± 3.3	13.8 ± 3.0	0.895
Trans-tricuspid gradient [cm/s]	2.06 ± 0.43	2.11 ± 0.52	2.00 ± 0.32	0.443
RVFW [mm]	4.10 ± 0.66	4.17 ± 0.69	4.01 ± 0.61	0.440
RVOT [mm]	26.2 ± 4.7	26.1 ± 5.1	26.2 ± 4.2	0.963
sPAP [mm Hg]	28.3 ± 6.5	29.9 ± 6.8	26.6 ± 5.8	0.124
mPAP [mm Hg]	23.1 ± 7.5	23.4 ± 8.2	22.7 ± 6.6	0.757
PVR [Wood units]	1.21 ± 0.26	1.24 ± 0.26	1.19 ± 0.24	0.524
Left ventricular ejection fraction [%]	70.6 ± 8.1	70.5 ± 8.9	70.8 ± 7.3	0.336
LLS	1.53 ± 2.22	0.7 ± 1.1	2.5 ± 2.8	0.011
SaO_2_ [%]	89.9 ± 5.3	92.6 ± 2.2	86.7 ± 6.2	0.004
ADMA [μmol/L]	0.70 ± 0.12	0.67 ± 0.10	0.74 ± 0.13	0.076
SDMA [μmol/L]	0.62 ± 0.10	0.59 ± 0.09	0.64 ± 0.11	0.141

All measurements were taken at high altitude at month 6 (M6). Abbreviations: RAA, right atrial area; RVFW, right ventricular free wall thickness; RVOT, right ventricular outflow tract; sPAP, systolic pulmonary arterial pressure; mPAP, mean pulmonary arterial pressure; PVR, pulmonary vascular resistance; LLS, Lake Louise Score; ADMA, asymmetric dimethylarginine; SDMA, symmetric dimethylarginine. Values are mean ± standard deviation. *p denotes the *p*-value for statistical comparison between well and poorly acclimatized subjects, respectively (two-tailed *t*-test).

The mean of mPAP in the echocardiography subgroup (*N* = 43) of our study was (mean ± SD) 23.1 ± 7.5 mm Hg; 18 participants (42%) showed an mPAP greater than 25 mm Hg (mean ± SD, 30.4 ± 3.9 mm Hg), out of which 9 (21%) were classified as HAPH (mPAP ≥ 30 mm Hg; mean ± SD, 33.9 ± 2.2 mm Hg). mPAP did not significantly correlate with hemoglobin concentration (*R* = 0.104; *p* = n.s.); nor was there a significant difference in mPAP between current smokers (23.5 ± 7.4 mm Hg) and nonsmokers (22.3 ± 8.2 mm Hg).

Morphological changes of the right ventricle were observed in nine study participants (20.9%). In four subjects, the diameter of the right ventricular outflow tract (RVOT) was 35 mm or larger, six subjects had right ventricular free wall thickness (RVFW) sizes of 5 mm or larger, and five subjects had a right atrial area at or above 18 mm.

### Predictive Role of Asymmetric Dimethylarginine and Symmetric Dimethylarginine for Pulmonary Arterial Pressure During Chronic Intermittent Hypobaric Hypoxia

**Figure 3:**
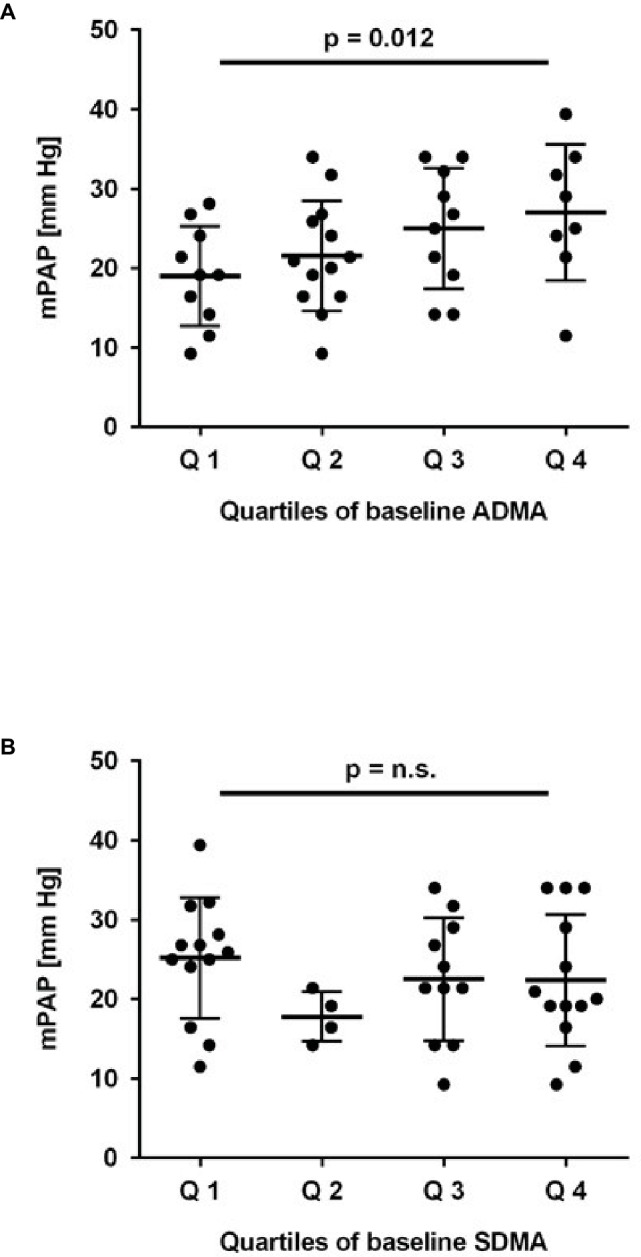
Association of baseline ADMA and SDMA with mean pulmonary arterial pressure (mPAP). Mean pulmonary arterial pressure was estimated by echocardiography after 6 months of chronic intermittent hypobaric hypoxia; it increased significantly with quartiles of baseline ADMA **(A)** but not SDMA **(B)**. *p* for linear trend was determined by one-way ANOVA.

To test the prospective association of ADMA and SDMA at baseline with the incidence of elevated mPAP at month 6, we compared mPAP in study participants stratified according to quartiles of baseline ADMA and SDMA concentration, respectively. There was a highly significant trend toward higher mPAP at month 6 for study participants who had a higher ADMA concentration at baseline (*p* = 0.012; Figure 3A), but no significant difference for mPAP stratified by baseline SDMA (Figure 3B).

Subsequently, we performed univariate logistic regression analysis for mPAP. Tertiles of baseline ADMA were significantly associated with mPAP at month 6 (*R* = 0.413; *p* = 0.007). Significance of the association was retained when quartiles of baseline ADMA were used (*R* = 0.389; *p* = 0.012). By contrast, tertiles of SDMA were not significantly associated with mPAP at month 6 (*R* = 0.081; *p* > 0.5). In multivariate regression analysis including systolic and diastolic blood pressure, SaO_2_, and hematocrit as traditional risk factors for HAPH, ADMA remained significantly associated with mPAP (*p* = 0.006). The change in ADMA concentration during 6 months of CIH was not significantly associated with mPAP (*R* = 0.306; *p* = 0.16). Furthermore, current smoking did not significantly affect the prospective association of ADMA with mPAP (*p* > 0.2).

**Figure 4:**
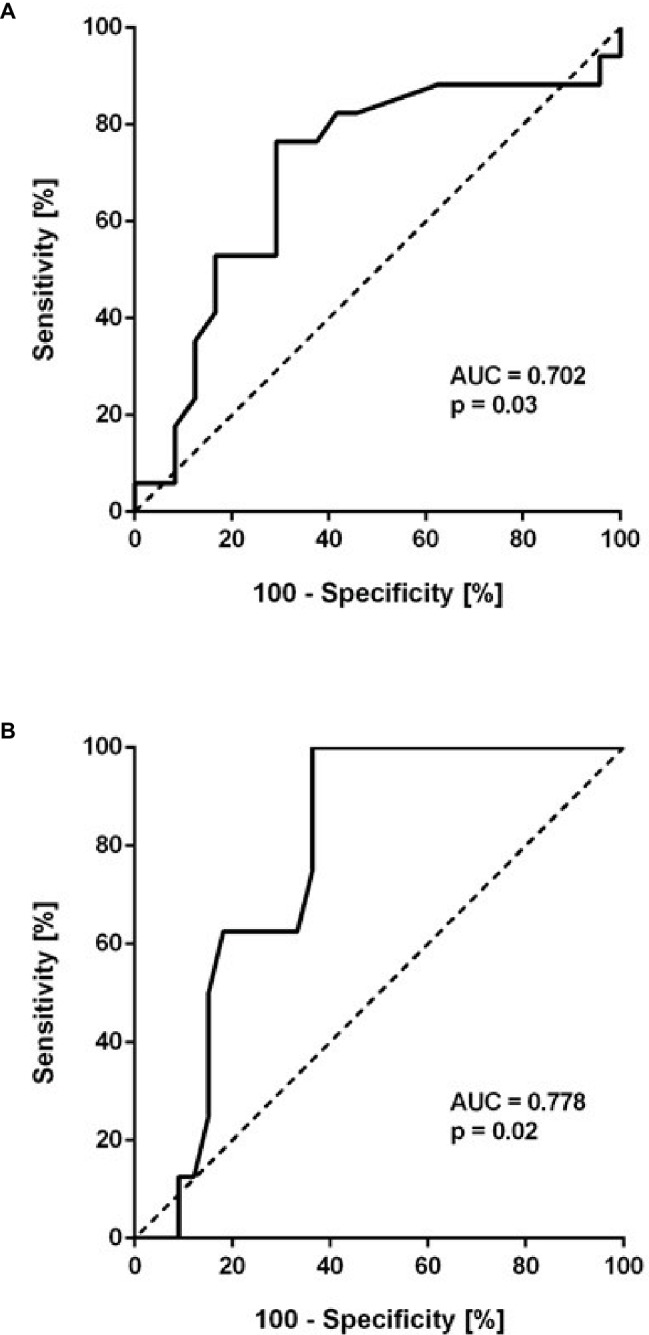
Receiver-operated curve (ROC) analysis of baseline ADMA (μmol/L) as a predictor of mean pulmonary arterial pressure (mPAP) > 25 mm Hg **(A)** and > 30 mm Hg **(B)**. The former cutoff is the usual threshold for the definition of pulmonary hypertension at sea level, the latter is the cutoff used at high altitude.

**Figure 5:**
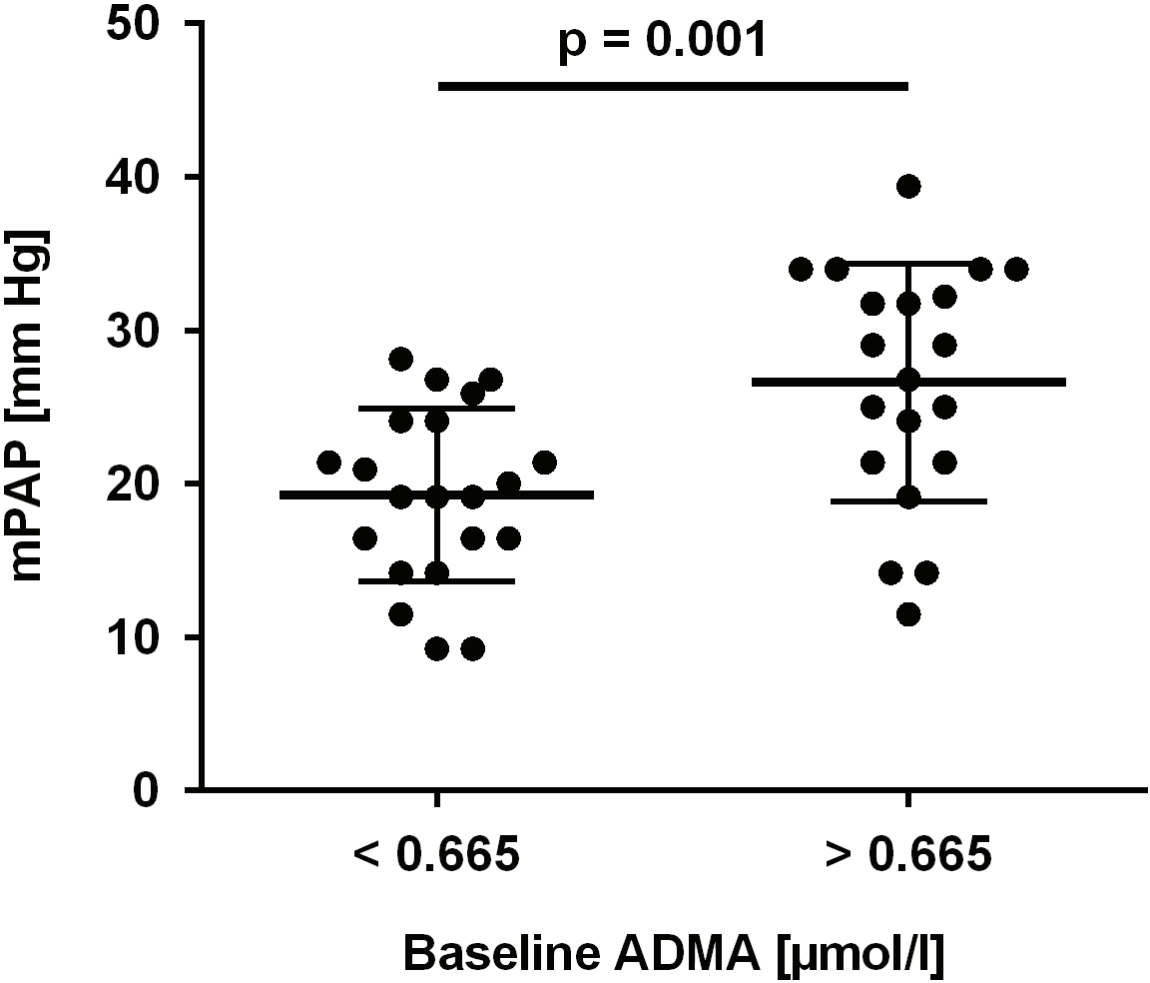
Mean pulmonary arterial pressure (mPAP) in study participants with baseline ADMA concentration below or above 0.665 μmol/L, the optimal cut-off value determined in ROC analysis.

To further determine a cut-off level for baseline ADMA to predict HAPH, ROC analyses were performed. Baseline ADMA showed a significant association with mPAP > 25 mm Hg at month 6 (AUC = 0.702; 95% CI, 0.530–0.875; *p* = 0.03; Figure 4A). Despite the small number of patients, baseline ADMA was also significantly associated with mPAP > 30 mm Hg, the cutoff for pulmonary hypertension at altitude (AUC = 0.778; 95% CI, 0.638–0.919; *p* = 0.02; Figure 4B). A baseline ADMA concentration of 0.665 μmol/L was the best prospective discriminator between subjects who developed HAPH or not (sensitivity 100, 95% CI, 63.1–100.0%; specificity 63.6, 95% CI, 45.1–79.6%). Accordingly, study participants with baseline ADMA concentration > 0.665 μmol/L had a significantly higher mPAP than those with baseline ADMA concentration ≤ 0.665 μmol/L (19.3 ± 5.6 vs. 26.6 ± 7.7 mm Hg, *p* = 0.001; Figure 5).

## Discussion

This is the first study to show that baseline ADMA (determined at sea level) predicts the presence of HAPH after 6 months of CIH. CIH had a selective impact on ADMA, while SDMA plasma concentration decreased and L-arginine concentration showed no specific pattern. ADMA was correlated to hematocrit, but it was unrelated to acute mountain sickness or the Lake Louise Score.

Our data reflect the difference in pathophysiology between acute responses to high altitude, such as AMS and sleeping disorders, and chronic responses to high altitude-associated hypoxia in the lung, especially HAPH. We observed a strong tendency of our study participants to cope with the repetitive stress of ascent to high altitude, as the prevalence of AMS in the morning after ascent was significantly lower at 4 and 6 months than initially and the Lake Louise Score decreased during the study. This finding is in line with previously published data for lowland Chilean mining workers (Richalet et al., 2002) and lowland Chinese workers (Wu et al., 2009), who were subjected to long-term CIH. By contrast, ADMA levels continuously increased during the duration of the study and were significantly related with HAPH, but not with AMS and the Lake Louise Score.

ADMA was discovered in the 1970s as one of several methylated L-arginine analogs that can be isolated from human plasma and urine (Ogawa et al., 1989). In 1992, evidence was provided that ADMA is an endogenous inhibitor of NO synthesis (Vallance et al., 1992b) and that it may accumulate in chronic renal failure (Vallance et al., 1992a). This observation was later extended to other cardiovascular conditions, and ADMA was identified as a prospective risk marker for major cardiovascular events and for total mortality in chronic renal failure (Zoccali et al., 2001), in patients with cardiovascular disease (Böger, 2006), in patients with idiopathic pulmonary arterial hypertension (Kielstein et al., 2005), and in the general population (Böger et al., 2009). By contrast, SDMA does not directly inhibit NO synthesis but it may interfere with cellular L-arginine uptake by cationic amino acid transporters (Closs et al., 1997). SDMA was initially proposed to be a marker renal function as it appeared to be biologically rather inert and its levels show a close correlation with glomerular filtration rate, but some studies found SDMA to be associated with cerebrovascular disease (Schulze et al., 2010; Lüneburg et al., 2012).

In the present study, we show a small but highly significant increase in systemic plasma ADMA concentration. Previously, we had observed a similar trend in a smaller study with a duration of only 3 months (Lüneburg et al., 2017). In our current study, baseline ADMA concentration was closer to published reference levels of healthy populations, in which the same analytical method as presently was used: in the Framingham Offspring cohort, mean plasma ADMA was calculated as 0.52 ± 0.11 μmol/L (2.5th and 97.5th percentile, 0.311–0.732 μmol/L) (Schwedhelm et al., 2009). This compares to 0.68 ± 0.11 μmol/L (2.5th and 97.5th percentile, 0.480–0.896 μmol/L) in the present study. It is unknown whether ethnic differences may explain this difference. Alternatively, the relatively high prevalence of overweight and obesity in our study cohort (25% with body mass index between 25 and 30, 12% with body mass index > 30) may have contributed to a higher mean ADMA (McLaughlin et al., 2006). Smoking is also known to affect ADMA concentration; our group previously reported smokers from a European population-based cohort to have lower ADMA than nonsmokers (Maas et al., 2007), although other investigators found the opposite association in a Chinese cohort of patients with early-onset coronary artery disease (Xuan et al., 2017), and a recent experimental study suggested that cigarette smoke increased ADMA in mouse lung tissue (Staab et al., 2015). The latter observations are in line with our current finding of lower baseline ADMA in nonsmokers than in smokers. However, the difference in ADMA between smokers and nonsmokers was no longer significant after 6 months of CIH, and smoking status did not significantly affect the association of ADMA with mPAP. The small absolute differences in systemic plasma ADMA and SDMA that we observed during CIH may relate to the fact that the major hypoxia-induced changes in dimethylarginine metabolism occur in the lungs, while systemic levels of dimethylarginines are a reflection of mixed blood from all tissues.

ADMA and SDMA are products of protein arginine methylation, which is a process of posttranslational protein modification catalyzed by a family of protein arginine methyltransferases [PRMT; for review cf. (Nicholson et al., 2009)]. Type I PRMTs asymmetrically methylate arginine residues, while type II PRMTs symmetrically methylate arginine, resulting in the release of ADMA and SDMA during the proteolysis of methylated proteins, respectively. ADMA is metabolized by dimethylarginine dimethylaminohydrolase (DDAH) while SDMA is not (Ogawa et al., 1989). Recent data suggest that both dimethylarginines are metabolized by alanine glyoxylate transferase-2 (AGXT2) (Rodionov et al., 2010; Lüneburg et al., 2014), although this enzyme was reported to have a lower affinity to dimethylarginines than DDAH and may thus serve as a “back-up” enzymatic pathway for ADMA when DDAH expression and/or activity is impaired (Rodionov et al., 2014).

In an experimental rat model of CIH and CH, we demonstrated elevation of ADMA but not SDMA, which is in line with our current results in humans. In the rat lung, elevated ADMA concentration was accompanied by increased oxidative stress as well as reduced DDAH activity (Lüneburg et al., 2016). Two distinct isoforms of DDAH have been identified in human tissues (Leiper et al., 1999). DDAH1 expression is reduced in chronic hypoxia (Millatt et al., 2003), and DDAH2 is inactivated by oxygen radicals (Lin et al., 2002). Intriguingly, DDAH1 knockout impairs NO-dependent vasodilation and enhances phenylephrine-induced pulmonary arterial vasoconstriction in mice (Leiper et al., 2007). Impaired NO-dependent vasodilation also occurs in humans exposed to acute or chronic hypoxia (Calderon-Gerstein et al., 2017; Lewis et al., 2017). As DDAH metabolizes ADMA but not SDMA, hypoxia-induced reduction of DDAH expression and/or activity might explain the increase in ADMA concentration in our study. However, changes in DDAH activity alone would not explain the reduction in SDMA concentration that we observed. Therefore, we speculate that the discrepant trends in ADMA and SDMA observed here may be explained by a compensatory increase in AGXT2 activity in the presence of decreased DDAH activity. This hypothesis would also explain the relatively small absolute change in ADMA concentration.

Another cause for the opposite trends of ADMA and SDMA plasma concentrations during CIH might be a discordant regulation of type I and type II PRMTs. PRMTs are highly expressed in lung tissue (Bulau et al., 2007). There is evidence that chronic hypoxia upregulates PRMT2 expression and elevates ADMA concentration (Yildirim et al., 2006). The changes in expression and activity of DDAH, AGXT, and PRMT isoforms under hypoxic conditions should be further examined in experimental models to clarify this pathophysiology.

It remains unclear why L-arginine concentration increased early in our study and then almost returned to baseline levels in the further course. L-arginine plasma concentration is influenced much more by the diet than dimethylarginine levels are. As we did not control for diet but participants were introduced to standardized canteen food at the beginning of the study, we cannot reliably interpret L-arginine concentrations in this study. Therefore, we did not calculate and interpret L-arginine/ADMA ratio, which is a biomarker for NO synthase substrate availability and a risk marker of mortality (Böger et al., 2009).

We performed echocardiography in a subgroup of study participants at the extremes of good and poor acclimatization to high altitude. Participants were selected based upon the presence of AMS at month 6 and/or SaO_2_ < 89% (poor acclimatization) or absence of AMS plus SaO_2_ > 89% (good acclimatization). By this means, we were able to compare individuals with good and poor acclimatization to high altitude; however, we found no significant differences in echo parameters between both groups. Rather, the echocardiography subgroup turned out to be well representative for the study group as a whole in terms of their baseline parameters and their ADMA and SDMA levels. While our study was ongoing, the Lake Louise Score was revised; sleep perturbance was omitted as a criterion in the revised Lake Louise 2018 Score (Roach et al., 2018). The Lake Louise 1993 Score, on which subject selection for echocardiography in our study was based, correlated very closely with the 2018 score (Pearson’s *R* = 0.966; *p* < 0.001).

As CIH represents a condition with significant periods of both, high altitude and sea level exposure, we have used both the cut-off value for mPAP at high altitude (i.e., 30 mm Hg) and the one used at sea level (i.e., 25 mm Hg) to discriminate between individuals with and without HAPH. ADMA showed a robust association with HAPH for both cut-off values. As hemoglobin concentrations in the participants of our study did not show excessive erythrocytosis, we believe that, specifically, the association of sea level ADMA with mPAP at high altitude reported here represents a reliable estimate of the role of ADMA for HAPH.

While several conditions have been characterized that help to predict the occurrence of AMS [for review cf. (Luks et al., 2017)], HAPH has remained difficult to predict. Similarly, therapeutic options like carboanhydrase inhibitors and corticosteroids have been tested in clinical trials for the prevention and treatment of AMS (Luks et al., 2017), but there still are only few therapeutic options to treat HAPH beyond oxygen administration and descent to low altitude (Villafuerte and Corante, 2016; West, 2017). Our study proposes a novel predictive marker for HAPH and, based on pathophysiological considerations, suggests a possible intervention strategy to prevent and/or treat HAPH. ADMA is a competitive inhibitor of NO synthesis; it displaces L-arginine from the substrate binding moiety of the enzyme, causing endothelial dysfunction, pulmonary vasoconstriction, and, finally, pulmonary hypertension. Experimental and clinical studies have shown that elevating the availability of substrate for NO synthase by dietary supplementation with L-arginine may be a means to revert pathophysiological effects of NO synthase inhibition (Böger, 2004).

It is tempting to speculate that our study may have practical implications for early diagnosis and for prevention of HAPH; ADMA may be a biomarker that adds to currently used diagnostic strategies in identifying individuals at high risk of developing HAPH. Further, supplementation with L-arginine may be an easy and safe means of intervention to prevent HAPH. Therefore, supplementation of individuals who have to subject themselves to chronic intermittent or chronic high altitude-associated hypoxia may be a novel intervention to reduce the incidence of HAPH. Controlled clinical trials are warranted to test this hypothesis in the future.

Our study has several limitations. Firstly, echocardiography data were available for the final analysis only in a subgroup of study participants, and no baseline echocardiographic assessment was performed. Based on data from our previous study that showed a correlation of ADMA with AMS (Lüneburg et al., 2017), we selected participants with good or poor acclimatization in order to highlight differences in echo parameters between these two extremes of clinical presentation. We could not confirm the previous correlation; but despite the small number of individuals, we found a highly significant predictive value of baseline ADMA for mPAP at month 6. Secondly, as the association of HAPH with ADMA is driven by a small number of individuals with HAPH (*N* = 9); this finding needs confirmation in future studies. Furthermore, our study is limited in that we did not control for diet, which might explain the large variability in L-arginine plasma concentration in this study and makes interpretation of L-arginine plasma concentration difficult. Finally, our study exclusively comprised young, healthy male army draftees; therefore, our findings may be limited to this group of individuals.

In conclusion, our study provides the first prospective evidence that ADMA is a risk marker of HAPH. Clinical scores and traditional risk markers that help to predict the incidence of acute mountain sickness do not help to predict individuals who develop HAPH. A cut-off level of 0.665 μmol/L, which is slightly lower than the cut-off level for ADMA to predict major adverse cardiovascular events and mortality in the general population (i.e., 0.70 μmol/L), has an excellent sensitivity and sufficient specificity to be used as a clinical marker.

## Ethics Statement

This study was carried out in accordance with the recommendations of the Declaration of Helsinki of the World Medical Association with written informed consent from all subjects. All subjects gave written informed consent in accordance with the Declaration of Helsinki. The protocol was approved by the Ethical Committee of Universidad Arturo Prat, Iquique, Chile.

## Author Contributions

PS, JB, JC, and FL-V conceived and designed the study. PS, JB, JC, ES, EP, RB, and JH performed data acquisition, analysis, and interpretation of the results. PS, JB, RB, and JH drafted the manuscript. JC, FL-V, ES, and EP critically revised the manuscript. All authors approved the final manuscript.

### Conflict of Interest Statement

The authors declare that the research was conducted in the absence of any commercial or financial relationships that could be construed as a potential conflict of interest.
